# Correlations Between ASSR Based on Narrow-Band CE^®^ Chirp, Click ABR, and Tone-Burst ABR in Audiological Evaluation of Children Under Anesthesia

**DOI:** 10.3390/life15060860

**Published:** 2025-05-27

**Authors:** Karolina P. Sideri, Laura F. Chiriboga, Piotr Henryk Skarzynski, Magdalena Beata Skarzynska, Milaine D. Sanfins, Maria F. Colella-Santos

**Affiliations:** 1 School of Medical Sciences, State University of Campinas, Campinas 13083-887, SP, Brazil; k102951@g.unicamp.br (K.P.S.); mfcolell@unicamp.br (M.F.C.-S.); 2 Health, Interdisciplinary Practice and Rehabilitation Program, University of Campinas, Campinas 13083-887, SP, Brazil; l207391@g.unicamp.br; 3 Department of Teleaudiology and Screening, World Hearing Center, Institute of Physiology and Pathology of Hearing, 05-830 Kajetany, Poland; p.skarzynski@csim.pl; 4 ENT Department, Maria Curie-Sklodowska University, 20-031 Lublin, Poland; 5 Center of Hearing and Speech Medincus, 05-830 Kajetany, Poland; m.skarzynska@csim.pl; 6 Department of Otolaryngology, Institute of Sensory Organs, 05-830 Warsaw, Poland; 7 Heart Failure and Cardiac Rehabilitation Department, Medical University of Warsaw, 02-091 Warsaw, Poland; 8 World Hearing Center, 05-830 Kajetany, Poland; 9 Department of Pharmacotherapy and Pharmaceutical Care, Pharmaceutical Department, Medical University of Warsaw, 02-091 Warsaw, Poland; 10 Institute of Sensory Organs, 05-830 Kajetany, Poland; 11 Department of Speech-Hearing-Language, Universidade Federal de São Paulo, São Paulo 04044-020, SP, Brazil; 12 Post-Graduate Program in Clinical Audiology, Instituto de Ensino e Pesquisa Albert Einstem, São Paulo 05652-000, SP, Brazil

**Keywords:** evoked potentials, auditory threshold, hearing, child, electrophysiology

## Abstract

Hearing plays a significant role in children’s development. The Auditory Steady State Response (ASSR) using a narrow band CE^®^ chirp is a technique that allows multiple stimuli to be presented simultaneously, making it possible to obtain electrophysiological thresholds with frequency specificity. The objective of this work is to analyze the findings obtained with the ASSR NB-CE^®^ chirp technique and compare them with two other methodologies—click ABR and tone-burst ABR—in the audiological assessment of children carried out under inhalation anesthesia. All the exams were performed in a single session. This study involved 71 children aged between 14 and 59 months, male and female, who were referred for ABR and ASSR due to suspected hearing loss and/or delay in speech/language development. Pearson’s correlation coefficient between the uncorrected and corrected thresholds obtained in the three methods demonstrated high correlations for all frequencies evaluated.

## 1. Introduction

Hearing is fundamental for a child’s development and is important for the more effective integration of the child into society, where oral communication predominates. Therefore, for children to develop oral language and acquire speech, it is essential that they have access to auditory stimuli [1]. It is also known that the central auditory nervous system has significant neural plasticity when stimulated early, which allows for an increase in neural connections and stimulation of auditory pathways [1,2,3]. Any interruption in this process leads to important functional losses that can impact the child’s quality of life, in which case, identification and effective intervention are required.

Issues relating to hearing assessment methodologies in childhood are still widely discussed. One way to assess hearing and predict thresholds in children electrophysiologically is the Auditory Brainstem Response (ABR) test using click stimuli, which is still considered the most clinically useful method [4,5,6]. Although the benefits of using ABRs to estimate hearing thresholds are well documented, the results obtained are limited by a number of factors: conditions for good recording related to the patient’s condition; restriction of the maximum stimulus presentation level (which with most equipment is 90 dB HL (hearing level); and finally, subjective visual analysis of the waves must be conducted to determine the electrophysiological threshold according to the characteristics of the click stimulus [4,7].

A click is a stimulus that is characterized as broadband. Applying the stimulus click on the ABRs allows a specific stimulation of the auditory system with an optimal nerve fiber synchronization over the 2–4 kHz frequency range for intensities close to the electrophysiological threshold [2,5]. That means it does not allow the collection of frequency-specific responses [4]. To compensate for the limitations of a click stimulus, the ABR using a tone-burst (TB) stimulus is considered the gold standard in child audiological assessment. It has been used to estimate a subject’s audiometric curve and can provide more accurate results for selecting and adapting hearing aids. It can help in better rehabilitating children with hearing loss, as it allows responses at specific frequencies of 500, 1000, 2000, and 4000 Hz [5].

However, the responses obtained with TB-ABR, especially at low frequencies and/or weak intensities, are more difficult to identify through visual analysis, which can lead to misinterpretations. Another difficulty with TB-ABR is related to the long time required to capture responses, as recording needs to be conducted for each ear and at one frequency and intensity at a time. Moreover, replications of tracings are needed at the lowest intensities to confirm that the electrophysiological threshold responses are valid [5].

An alternative to obtaining electrophysiological thresholds with frequency specificity is the use of the Auditory Steady State Response (ASSR), in which stimuli are presented simultaneously in both ears, at four different frequencies and possibly different intensities. In this way, responses can be detected automatically based on algorithms and statistical analyses built into the equipment [8,9].

Several studies have aimed to verify ASSR assessments and facilitate their use in clinical practice. The most recent ones have discussed the results of ASSR using a chirp stimulus, especially the Narrow Band CE^®^-chirp (NB CE^®^-chirp) developed by Elberling [10]. The NB CE^®^-chirp is designed to combine the advantages of wave compensation on the basilar membrane, which a chirp stimulus provides, with frequency specificity [9,11,12]. The general aim is to improve detection rates and reduce testing time.

In terms of the necessary conditions needed for recording, it is known that the patient must remain completely relaxed and without any type of body movement during the exam [7]. For the pediatric population, the available alternatives are to carry out the examination during natural sleep or, when required, with anesthetic assistance. However, few studies have described the audiological findings of ASSR in childhood populations or even the need to use anesthesia, which is often justified by the difficulties encountered in performing auditory evoked potentials in this age group [13].

Taking all these considerations together, this work aims to analyze the electrophysiological thresholds obtained using the ASSR with Narrow Band CE^®^-chirp stimulus and compare them to findings of the ABR with click and tone burst stimuli. The test subjects were children aged between 14 and 59 months, and the experiments were performed under inhalation anesthesia.

## 2. Materials and Methods

This study was approved by the Research Ethics Committee under process No. 4.468.092/2020. The guardians responsible for the research participants were invited and informed about the study and consented to participate by signing a Free and Informed Consent Form. Data collection took place prospectively between the periods of January and November 2021.

### 2.1. Participants

The sample consisted of 71 children aged between 14 and 59 months (average age of 32.25 months), 62 male and 9 female, who were medically referred for ABR and ASSR due to suspected hearing loss and/or delay in speech/language development. Cases that presented external and/or middle ear malformations, syndromes, and suspected cases of Auditory Neuropathy Spectrum were excluded.

After agreeing to participate in the research and prior to carrying out the exams, the guardians briefly answered some questions about the subjects—full name of the guardian, full name and date of birth of the child, and by which professional the exam was requested—and the reasons behind their referral for audiological evaluation. The exams themselves were performed in a surgical room located on the hospital premises and conducted under inhalation anesthesia in a single session varying from 40 to 60 min. The assessments were carried out by an audiologist with expertise in the area, with the assistance of an anesthetist responsible for administering the inhaled sedative.

### 2.2. General Procedures

Upon admission of the patient into the operating theatre, anesthesia was induced using an inhalational anesthetic (Sevoflurane). Then the audiologist cleaned the patient’s skin with an abrasive paste and positioned disposable surface electrodes, with the active vertex and ground electrodes located on the forehead and in the Fz and Fpz positions, respectively, and the reference electrodes on the right and left mastoids. Each electrode had an impedance of <5 kΩ and an inter-electrode impedance of <2 kΩ. Insert headphones (ER-3A) were used and the equipment used for all exams was the Eclipse EP 25 software version 4.6.0.33 (Interacoustics).

#### 2.2.1. Click ABR Procedures

The parameters used to record the click ABR were click stimuli presented monaurally with rarefied polarity. Responses were low-pass filtered at 3000 Hz and high-pass filtered at 100 Hz, with a total recording of 2000 stimuli at a presentation rate of 37.7 stimuli/s and an analysis window of 15 ms. To verify the integrity of the auditory pathway, the presence and absolute latency of waves I, III, and V at 80 dBHL were analyzed, as well as the interpeak latencies I–III, III–V, and I–V. To measure electrophysiological threshold, the presence or absence of wave V at decreasing intensities, and its absolute latency, were also analyzed. The recordings made at 80 dBHL, as well as those at the electrophysiological threshold, were replicated to verify reproducibility [13].

#### 2.2.2. Tone Burst ABR Procedures

The parameters used to record the TB-ABR were tone burst stimuli presented monaurally at frequencies of 4000, 2000, 1000, and 500 Hz using alternating polarity, a low-pass filter of 1500 Hz and a high-pass filter of 33 Hz, recording a total of 2000 stimuli for each intensity and frequency. The presentation rate was 35.1 stimuli/s, and the analysis window was 20 ms. The presence, absolute latency, and reproducibility of wave V at its lowest recorded intensity were analyzed to detect the electrophysiological threshold. The correction of electrophysiological thresholds as proposed by Stapells [14] was applied.

#### 2.2.3. ASSR Procedures

The stimuli used to record ASSR were NB CE^®^-chirps, presented binaurally and simultaneously, at frequencies of 500, 1000, 2000, and 4000 Hz, with a modulation frequency of 90 Hz. The response detection algorithm adopted was “Speed”, and the significance value adopted was *p* ≤ 0.05. The thresholds obtained in ASSR were specified as dBHL and subsequently corrected with the correction factors applied by the manufacturer’s algorithm, giving the estimated thresholds as dBeHL (estimated hearing level).

### 2.3. Statistical Analysis

For statistical analysis, the significance value adopted was ≤5% (*p* ≤ 0.05). SPSS Statistics software, version 28.0 (IBM Corp., Armonk, NY, USA), was used. To compare the ears, Student’s *t*-test was used as a parametric test for paired samples. Calculations relating to the Student’s *t*-test were performed using a bias-corrected and accelerated bootstrap sampling method based on 1000 samples. Bonferroni correction was used for multiple comparisons. The effect size of the difference between ears was measured using the *d* coefficient [15].

Pearson’s correlation coefficient (*r*) [16] was used to evaluate the correlation between the click ABR, TB-ABR, and ASSR. Calculations of the correlation coefficient, 95% confidence intervals, and *p*-value were carried out using the bias-corrected and accelerated bootstrap sampling method based on 2000 samples. To interpret the coefficient *r*, the following criteria were adopted: small—between |0.100| and |0.299|; medium—between |0.300| and |0.500|; high—above |0.500|.

A linear mixed model was used to investigate the influence of sex, age group, and reason for referral on the thresholds obtained in the click ABR, TB-ABR, and ASSR. This technique is an analysis that measures correlations between multiple factors and can include random effects, which is helpful in understanding the phenomenon under investigation [16]. Furthermore, it is a robust technique resistant to violations of the assumptions traditionally assumed in parametric analyses [17].

To control for individual variability, individuals were regarded as random effects on the intercepts of each model. Variables relating to sex, age group, and reason for referral were regarded as fixed effects. For the modeling of TB-ABR and ASSR, the tested frequencies and the conditions with and without correction were regarded as control variables. The significance of fixed effects was assessed with *F*-tests, with the number of degrees of freedom calculated using the Kenward–Roger method and effect size calculated by converting the *F*-statistic to an *r*-coefficient [16]. Variances were estimated using the Residual Maximum Likelihood technique in which an “unstructured” covariance structure was assumed.

## 3. Results

Table 1 presents the Pearson’s correlation coefficients between the thresholds obtained in the click ABR, TB-ABR, and ASSR listed according to frequency and considering the thresholds obtained without applying any correction factors. For comparison, Table 2 presents similar correlations between the thresholds, but this time using thresholds obtained after the application of the correction factors.

The results in Table 1 and Table 2 demonstrate that, for both uncorrected and corrected thresholds, high correlations were observed between procedures for all frequencies evaluated.

For all exams, there was a statistically significant and medium-sized effect in relation to the reason for referral. Small effects without statistical significance were observed for the factors of sex and age group (Table 3). These results indicate that, regardless of the other variables (frequency, application or not of correction to the threshold, sex, and age group), the reason for referral to the exam had a high effect on the determined thresholds.

To explore in more detail the effect of the reason for referral for testing, Table 4 presents post hoc pairwise comparisons according to reason using Student’s *t*-test (with Bonferroni correction for multiple comparisons) for each model. The effect size for each analysis was evaluated by converting the *t*-statistic to an *r*-coefficient [15].

The results in Table 4 demonstrate that, for all exams, the group with suspected hearing loss presented a statistically significant difference in mean effect size compared to the other groups, and, in all cases, the group with suspected hearing loss presented higher mean thresholds compared to the other groups. There was no statistically significant or significant difference between the groups complaining of speech/language delay and suspected autism.

## 4. Discussion

Many electrophysiological investigations have focused on improving how auditory evoked potentials are collected. The general aim has been to strengthen their use in evidence-based clinical practice. Electrophysiological methods have been widely applied in audiology because they objectively determine hearing thresholds, as well as give information about hearing threshold changes such as type, configuration, and degree. They allow interventions and rehabilitation to be conducted in cases where it is impossible to make assessments using subjective and behavioral measures.

Regarding the various types of ABR used to study the auditory pathway and obtain electrophysiological hearing thresholds, the most recent protocols recommend the use of click stimuli as the gold standard. Click stimuli can assess the integrity of the auditory pathways after which frequency-specific stimuli, such as a tone burst, can be used to ascertain electrophysiological thresholds (typically at 500, 1000, 2000, and 4000 Hz) [13].

TB-ABR using specific-frequency stimuli is commonly built into commercial equipment, and many studies have validated its clinical application [5,18]. The main clinical application of TB-ABR is aimed at determining children’s electrophysiological thresholds at various frequencies, often used when the responses obtained through behavioral or tonal audiometry are not sufficiently reliable [7,19].

However, due to the conditions necessary to carry out a TB-ABR exam, assessments using frequency-specific stimuli often encounter limiting factors that make assessment difficult. In particular, to record auditory evoked potentials, it is essential that the subject remain still and relaxed. For children, this means that the examination is better carried out with the child in natural sleep. However, it is not always feasible for an exam to be carried out in natural sleep due to other circumstances—the need for skin hygiene, presentation of stimuli at high intensities, and time to complete the exam—meaning that in some cases anesthesia is necessary. Previous studies on the effects of anesthesia on ABR, especially Sevoflurane, have indicated that there may be an increase in wave V latency and thus an increase in the I–V interpeak interval [20]. However, such a slight increase has little or no effect on electrophysiological thresholds, so it remains a viable sedation method when evaluating children. Furthermore, for ASSR with 90 Hz modulation, there is evidence that sedation states do not affect responses [9,21].

Regarding the time needed to perform an ABR at a specific frequency (TB-ABR in particular), a full examination can take a long time. In order to guarantee a valid response, it is recommended to evaluate four airborne frequencies and intensities (by air) in each ear, as well as replicate the recording at the lowest observable intensity of wave V. In some cases, bone-based assessments are also necessary, further increasing examination time. In assessments carried out in natural sleep, more than one session may be necessary to complete the examination, prolonging the time and making diagnosis difficult. In some cases, the whole examination needs to be abandoned [5,7].

From this perspective, new resources such as the ASSR have been developed with the aim of estimating hearing thresholds in cases where the assessment of behavioral responses is difficult. The ASSR also reduces evaluation time since multiple stimuli can be presented binaurally [11,21]. Combining ASSR with an NB CE^®^-chirp stimulus provides extra benefits because it produces responses with greater amplitudes and reduced examination time [10,22].

When all the techniques available for objective hearing assessment are considered, additional work is still needed to define new guidelines for the best protocol to be used in assessing children. The main questions raised by recent studies concern the possibility of replacing the ABR using frequency-specific stimuli, such as tone burst, with the ASSR technique and to what extent the ASSR is broadly compatible with estimated behavioral thresholds [9,11,22].

Although other studies have already compared different techniques and populations [9,22,23,24,25,26,27], the context of the present research has been studies that have used ASSR assessments using the NB CE^®^-chirp stimulus, which, as previously mentioned, combines the advantage of wave compensation in the cochlea with frequency specificity, enabling even faster detection of the response in a shorter testing time.

In this study, we carried out a statistical analysis of the agreement between the different techniques using Pearson’s correlation coefficient, finding that high correlations were observed between all procedures for all frequencies evaluated. Other comparison studies have obtained similar correlation values [18,19,20,21,22].

There are several factors affecting the degree of correlation between the techniques, among which is the construction of the stimulus. In the case of the NB CE^®^-chirp, the main aim is to optimize the detection of ASSR responses [10]. Other possible effects relate to the correction factors (from dBHL to dBeHL), which for TB-ABR depend linearly on intensity, but for ASSR thresholds depend on intensity in a non-linear way [7,23].

Another important aspect when comparing techniques is the need for subjective assessment by an audiologist. In TB-ABR there is a subjective interpretation by the evaluator, who must look for the presence or absence of responses in the exam tracings, which can lead to interpretation bias [5]. Furthermore, due to the time required for a complete assessment using TB-ABR, testing is often carried out at a single standard intensity and frequency, although these might not necessarily correspond to the electrophysiological threshold [13].

On the other hand, because ASSR can present multiple binaural stimuli, the time advantage allows for additional testing at lower intensities [11]. This eliminates subjective bias since valid responses are computed by the equipment and do not depend on the audiologist’s subjective judgment [23]. In several studies [18,19,21], the authors considered the use of ASSR NB CE^®^-Chirp effective as a method to predict frequency-specific hearing thresholds in children.

Regarding the possible effects of sex and age group, the literature on click ABR points to effects on absolute latency and wave amplitude [24]. In children, changes as a function of age occur mainly during the first 18 months of life, when absolute latencies decrease and wave amplitudes increase, probably due to myelination of the cochlear nerve [25]. In relation to sex, effects are only seen in relation to absolute wave latency, especially in the adult and elderly population, where, in general, females present lower absolute wave latencies compared to males [24,25].

In the analysis presented here, it can be observed that sex and age group did not interfere with the thresholds obtained in the click ABR, TB-ABR, and ASSR. However, the reason for requesting an exam was shown to be significantly related to the observed thresholds, and, in cases where hearing loss was suspected, higher thresholds were observed compared to other reasons.

There are, however, some limitations that must be considered for the use and applicability of ASSR in clinical practice. Perhaps the biggest limitation is that not all commercial equipment that performs an ASSR has been adequately validated. ASSR systems vary considerably in the way they analyze responses, leaving room for greater variability in reported findings and making it difficult to obtain solid scientific evidence [23].

Another issue relates to the way in which ASSR responses are obtained and analyzed in the frequency domain, not in the time domain as in ABR. This means that some information regarding absolute latencies, amplitudes, and interpeak intervals is lost. For this reason, it is recommended that the ASSR not be used as the only method, and ideally, it should be carried out after an ABR [23,26,27].

Furthermore, the ASSR does not appear to be capable of evaluating cases of auditory neuropathy, as it may detect non-neural responses arising from artifacts, vestibular responses, or poor neural synchrony. Therefore, for cases in which auditory neuropathy is suspected, the recommendation is that ASSR should not be applied [23].

Finally, although ASSR can evaluate multiple frequencies, it is known that the detection of responses decreases for multiple presentations of frequencies from 65 dBHL onwards and should not be carried out simultaneously for intensities above 80 dBHL. Responses to stimuli with intensities above 100 dBHL may reflect responses of vestibular origin and must be analyzed with caution [23].

## 5. Conclusions

In this study, it was possible to analyze the thresholds obtained with frequency specificity using the TB-ABR and ASSR techniques, to compare them, and to verify agreement at frequencies of 500, 1000, 2000, and 4000 Hz. It was also possible to observe the effects of applying or not applying correction factors in the TB-ABR and ASSR. The variables of gender and age group did not influence the thresholds obtained, unlike the variable ‘reason for referral’.

Based on these findings, the ASSR with NB CE^®^-chirp stimulus proved to be useful in predicting the estimated hearing thresholds with frequency specificity in the child population in exams performed under inhalational anesthesia, contributing to greater knowledge about the hearing conditions of these subjects.

## Figures and Tables

**Table 1:** Analysis with frequency of the Pearson correlation coefficients between the uncorrected thresholds obtained in the click ABR, TB-ABR, and ASSR.

	Frequency							
	500 Hz		1000 Hz		2000 Hz		4000 Hz	
Agreement Analyzed	*r*	*p*	*r*	*p*	*r*	*p*	*r*	*p*
Click ABR × TB-ABR	0.972 [0.902, 0.990]	<0.001 *	0.959 [0.898, 0.976]	<0.001 *	0.968 [0.911, 0.982]	<0.001 *	0.985 [0.953, 0.994]	<0.001 *
Click ABR × ASSR	0.942 [0.862, 0.967]	<0.001 *	0.958 [0.878, 0.980]	<0.001 *	0.967 [0.919, 0.990]	<0.001 *	0.956 [0.851, 0.989]	<0.001 *
TB-ABR × ASSR	0.981 [0.934, 0.992]	<0.001 *	0.946 [0.833, 0.970]	<0.001 *	0.954 [0.885, 0.978]	<0.001 *	0.963 [0.864, 0.981]	<0.001 *

*r* = Pearson’s correlation coefficient; * = statistically significant at level of 5% (*p* ≤ 0.05).

**Table 2:** The same as for Table 1, but showing Pearson correlation coefficients after the thresholds have been corrected.

	Frequency							
	500 Hz		1000 Hz		2000 Hz		4000 Hz	
Agreement Analyzed	*r*	*p*	*r*	*p*	*r*	*p*	*r*	*p*
Click ABR × TB-ABR	0.972 [0.902, 0.990]	<0.001 *	0.959 [0.898, 0.976]	<0.001 *	0.968 [0.911, 0.982]	<0.001 *	0.985 [0.953, 0.994]	<0.001 *
Click ABR × ASSR	0.935 [0.830, 0.964]	<0.001 *	0.967 [0.899, 0.984]	<0.001 *	0.970 [0.925, 0.991]	<0.001 *	0.957 [0.848, 0.991]	<0.001 *
TB-ABR × ASSR	0.979 [0.939, 0.990]	<0.001 *	0.955 [0.862, 0.975]	<0.001 *	0.956 [0.911, 0.976]	<0.001 *	0.966 [0.894, 0.981]	<0.001 *

*r* = Pearson’s correlation coefficient; * = statistically significant at level of 5% (*p* ≤ 0.05).

**Table 3:** Tests of fixed effects of the linear mixed model to examine the influence of sex, age group, and reason for referral on the thresholds obtained by click ABR, TB-ABR, and ASSR.

Factor	Click ABR					TB-ABR					ASSR				
	*F*	df1	df2	*p*	*r*	*F*	df1	df2	*p*	*r*	*F*	df1	df2	*p*	*r*
Sex	2.491	1	65	0.119	0.192 ^†^	1.700	1	62,006	0.197	0.163 ^†^	2.551	1	66,050	0.115	0.193 ^†^
Age group	1.939	1	65	0.169	0.170 ^†^	2.357	1	62,101	0.130	0.191 ^†^	2.003	1	65,996	0.162	0.172 ^†^
Reason	7.027	2	65	0.002 *	0.422 ^††^	7.531	2	62,012	0.001 *	0.442 ^††^	6.751	2	65,993	0.002 *	0.412 ^††^

*r* = Pearson’s correlation coefficient; * = *p* ≤ 0.05; df = degrees of freedom; † = small effect; †† = medium effect.

**Table 4:** Post hoc analysis of the effect of reason for referral on thresholds in Click ABR, TB-ABR, and ASSR.

Exam	Comparison	Difference	SE	*t*	df	*p*	ES
Click ABR	Speech/language delay × Suspected autism	−0.73	3.39	−0.215	65	>0.999	0.027
	Speech/language delay × Suspected hearing loss	−14.76	4.01	−3.684	65	0.001 *	0.416 ^††^
	Suspected autism × Suspected hearing loss	−14.03	4.59	−3.061	65	0.010 *	0.355 ^††^
TB-ABR	Speech/language delay × Suspected autism	−0.27	2.89	−0.093	61,982	>0.999	0.012
	Speech/language delay × Suspected hearing loss	−12.84	3.40	−3.779	62,042	0.001 *	0.433 ^††^
	Suspected autism × Suspected hearing loss	−12.57	3.87	−3.249	62,021	0.006 *	0.381 ^††^
ASSR	Speech/language delay × Suspected autism	−0.37	3.41	−0.110	66,000	>0.999	0.014
	Speech/language delay × Suspected hearing loss	−14.76	4.13	−3.578	65,995	0.002 *	0.403 ^††^
	Suspected autism × Suspected hearing loss	−14.39	4.63	−3.109	65,975	0.008 *	0.357 ^††^

*t* = *t*-test with Bonferroni correction for multiple comparisons; * = *p* ≤ 0.05; SE = standard error of the difference/estimate; df = degrees of freedom; ES = effect size; ††: medium effect.

## Data Availability

The raw data supporting the conclusions of this article will be made available by the authors upon request due to privacy concerns.

## Abbreviations

The following abbreviations are used in this manuscript:

ASSR	Auditory Steady State Response
ABR	Auditory Brainstem Responses
TB-ABR	Tone Burst—Auditory Brainstem Responses
NB-CE^®^ chirp	Narrow-Band CE^®^ Chirp
dB HL	Decibel Hearing Level
Hz	Hertz
TB	Tone Burst
dB eHL	Decibel Estimated Hearing Level
